# Cooperative Interaction of Hyaluronic Acid with Epigallocatechin-3-O-gallate and Xanthohumol in Targeting the NF-κB Signaling Pathway in a Cellular Model of Rheumatoid Arthritis

**DOI:** 10.3390/antiox14060713

**Published:** 2025-06-11

**Authors:** Francesco Longo, Alessandro Massaro, Manuela Mauro, Mario Allegra, Vincenzo Arizza, Luisa Tesoriere, Ignazio Restivo

**Affiliations:** Department of Biological, Chemical and Pharmaceutical Sciences and Technologies (STEBICEF), University of Palermo, 90128 Palermo, Italy; francesco.longo03@unipa.it (F.L.); alessandro.massaro01@unipa.it (A.M.); manuela.mauro01@unipa.it (M.M.); mario.allegra@unipa.it (M.A.); vincenzo.arizza@unipa.it (V.A.); luisa.tesoriere@unipa.it (L.T.)

**Keywords:** hyaluronic acid, xanthohumol, epigallocatechin-3-o-gallate, inflammation, rheumatoid arthritis

## Abstract

Current intra-articular therapies with hyaluronic acid (HA) provide symptomatic relief in joint diseases, but have limited efficacy in counteracting oxidative stress and inflammation, key drivers of cartilage degradation in rheumatoid arthritis (RA). To address this limitation, the potential of combining HA with the phytochemicals xanthohumol (XAN) and epigallocatechin-3-O-gallate (EGCG), known for their antioxidant and anti-inflammatory properties, was evaluated in a cellular model of RA (SW982 synoviocytes stimulated with interleukin-1β, IL-1β). The Chou–Talalay method demonstrated that their combination synergistically reduced reactive oxygen species (ROS) and nitric oxide (NO) levels. The “TRIPLE” combination (HA + XAN + EGCG) showed the lowest combination index and the highest dose reduction index. Compared to individual treatments, TRIPLE significantly decreased IL-1β-induced IL-6, IL-8, TNF-α, and MMP-3 levels, while increasing the levels of the anti-inflammatory cytokine IL-10. Western blot analysis revealed a marked reduction in iNOS, COX-2, and MMP-3 protein expression following TRIPLE treatment. Moreover, the combination inhibited IL-1β-induced phosphorylation of IκB and p65, thereby preventing NF-κB activation. These findings suggest that integrating XAN and EGCG into injectable HA formulations may represent a promising strategy to improve the management of joint inflammation in RA.

## 1. Introduction

Hyaluronic acid (HA) is a natural glycosaminoglycan composed of repeating units of a disaccharide formed by D-glucuronic acid and N-acetyl-D-glucosamine. Synthesized by most cells in the vertebrate body, particularly by mesenchymal cells, this biopolymer is a crucial component of the tissue extracellular matrix [1]. The high-molecular-weight, linear, and unbranched chains of hyaluronan form a gel-like network that ensures tissue hydration and facilitates lubrication, particularly in highly specialized environments such as cartilage and synovial fluid [2]. This viscoelastic matrix plays a crucial role in joint function, contributing to the smooth movement of articulating surfaces and modulating the interactions between extracellular components and cellular elements, including hyaluronan-coated extracellular vesicles [3]. Here, it acts as a shock absorber for mechanical impacts and reduces friction between bone ends [4]. In addition to its role as a passive structural component of the extracellular matrix, HA actively participates in cell signaling by interacting with specific cell-surface receptors known as hyaladherins, such as CD44, which are widely expressed across various cell types [5]. Through these interactions, HA modulates key cellular processes, including the inhibition of inflammatory responses and the preservation of extracellular matrix integrity in chondrocytes exposed to catabolic stimuli like interleukin-1β [6]. Moreover, HA has been shown to have regenerative properties in both fibroblasts and keratinocytes, as well as moisturizing and anti-aging effects [7]. Beyond its structural role, HA exhibits notable antioxidant and immunomodulatory properties. These include the suppression of pro-inflammatory cytokine production and the promotion of anti-inflammatory mediators in immune cells and joint-resident cells, such as chondrocytes and synoviocytes [8]. These actions contribute to HA’s protective role in various physiological and pathological contexts, including skin aging, lung inflammation, and vascular and joint diseases [9], with high-molecular-weight HA shown to attenuate IL-6-induced matrix metalloproteinase expression by upregulating the ERK pathway inhibitor MKP-1 [10]. Rheumatic diseases, characterized by an inflammatory state of the joints, tendons, ligaments, and bones, cause substantial changes in the quantity and molecular weight of HA in synovial fluid, with significant implications for the severity and progression of the illness [11]. Rheumatoid arthritis (RA) is a long-lasting autoimmune condition marked by chronic inflammation of the synovial membrane, gradual deterioration of cartilage and bone, and various systemic effects [12]. It affects around 0.5–1% of the global population, with a higher incidence observed in women and individuals over the age of 40 [13]. Although biologic and targeted synthetic disease-modifying antirheumatic drugs (DMARDs) have significantly advanced treatment options, many patients still struggle to achieve long-term remission and may experience notable side effects. This highlights the ongoing need for safer, more comprehensive therapeutic approaches [14]. On a molecular level, RA development is fueled by an overactive immune response—both innate and adaptive—leading to an overproduction of pro-inflammatory cytokines such as IL-1β, TNF-α, and IL-6 [15]. In combination with elevated oxidative stress, these factors drive key pathological changes like synovial tissue overgrowth, extracellular matrix breakdown, and chondrocyte death [16]. In fact, under inflammatory conditions, cartilage tissue cells lose the ability to regulate the synthesis and degradation process of HA, and its roles as an antioxidant and modulator of the inflammatory response in joints are lost. Furthermore, under conditions of tissue injury or chronic inflammation, hyaluronan undergoes enzymatic degradation, leading to the accumulation of low-molecular-weight fragments within the joint microenvironment. In contrast to the native high-molecular-weight form, these fragments function as damage-associated molecular patterns [17], stimulating the release of pro-inflammatory mediators and promoting synovial hyperplasia and the progression of inflammatory joint diseases [18]. Among these mediators is the pro-inflammatory cytokine interleukin-1beta (IL-1β), which is locally synthesized by synoviocytes and chondrocytes. IL-1β is the main cytokine involved in rheumatoid diseases [19]. It has been shown to play a central role in cartilage or synovial fluid damage by disrupting the balance between degradation and repair processes [20].

Intra-articular administration of high-molecular-weight HA is a widely adopted therapeutic approach for patients with rheumatic and degenerative joint diseases [21]. This treatment not only provides mechanical support and viscoelastic supplementation to the joint, but also exerts anti-inflammatory and chondroprotective effects by modulating the local cellular environment [22]. This administration route, which allows the release of the polysaccharide directly into the affected joint, provides a localized therapeutic effect, reducing pain and generally improving joint function [23]. To enhance the therapeutic efficacy of HA, it is often co-administered with corticosteroids or non-steroidal anti-inflammatory drugs, either in free form or as part of advanced delivery systems [24]. This combinatorial approach has been shown to provide superior pain relief and improved clinical outcomes compared to HA alone, particularly in the management of knee osteoarthritis [25]. Moreover, recent advancements in HA-based formulations have focused on increasing resistance to enzymatic degradation and oxidative stress, further prolonging intra-articular residence time and therapeutic effectiveness [26]. Since even local corticosteroid treatments are associated with numerous side effects [27], and their clinical application is further limited by pharmacokinetic drawbacks, such as rapid clearance from the joint space [28], there is growing interest in the use of alternative therapeutic strategies that can be developed by associating HA with plant-derived molecules with proven bioactivity and without significant adverse consequences.

Epigallocatechin-3-O-Gallate (EGCG), a catechin found in green tea, and Xanthohumol (XAN), a prenylflavonoid found in hop inflorescence, are polyphenols that have been extensively studied for their antioxidant and anti-inflammatory activities. Both phytochemicals have shown beneficial individual effects in models of rheumatic diseases in both cells and animals, supporting their use in the treatment of these diseases. In particular, EGCG has been shown to counteract oxidative stress-induced dysfunction in human chondrocytes by activating the Keap1/Nrf2/ARE signaling pathway [29], and to suppress IL-1β-induced inflammatory responses [30] by downregulating pro-inflammatory cytokine production, chemokine release, and matrix metalloproteinase activation in both chondrocytes and synovial fibroblasts [31]. Moreover, intra-articular injections of EGCG ameliorate the symptomatology both in post-traumatic [32] and in aging-related osteoarthritis animal models [33]. It has been demonstrated that XAN can also effectively downregulate IL-1β-induced inflammation and extracellular matrix degradation in chondrocytes by modulating the HO-1/C/EBPβ signaling pathway [34]. In vivo, intraperitoneal administration of XAN was shown to alleviate chronic pain in a collagen-induced arthritis mouse model, likely through the suppression of mitochondrial-mediated inflammatory responses [35]. Furthermore, studies using human synovial sarcoma SW982 cells have contributed to the phenotypic characterization of inflammatory responses relevant to synovial pathophysiology, and support XAN’s therapeutic potential in joint inflammation [36].

To evaluate the potential use of natural molecules as adjuvants of HA in injectable pharmaceutical preparations, in this work, we investigated the combined protective effect of the polysaccharide with EGCG or/and XAN in a cell model of rheumatoid arthritis, and analyzed the eventual cooperation of the biomolecules in reducing the inflammatory cell response. Since different fractions of HA, characterized by different molecular weights, can have specific effects on cellular signaling, a high-molecular-weight HA preparation (3–3.5 × 10^6^ Daltons) was employed in this study. We used SW982 cells, a human synovial sarcoma cell line characterized by the expression of inflammation enzymes such as cyclooxygenase-2 (COX-2), inducible nitric oxide synthetase (iNOS), matrix metalloproteases (MMPs), and other mediators such as IL-6, IL-8, and tumor necrosis factor-alpha (TNF-α), in response to IL-1β. In addition, the signaling pathway leading to NF-kB activation, a pivotal nuclear transcription factor in mediating the inflammatory response and a target of the anti-inflammatory action of several phytochemicals [37], was explored to elucidate the molecular mechanisms underlying the observed effects. This study offers new evidence that combining high-molecular-weight HA with selected polyphenols produces synergistic effects in reducing inflammatory signaling in synoviocytes. These results pave the way for the development of advanced intra-articular formulations that harness the combined benefits of HA and phytochemicals, with the potential to enhance current treatments for joint inflammation. In this framework, HA, EGCG, and XAN emerge as a promising trio for restoring joint homeostasis and protecting cartilage health.

## 2. Materials and Methods

### 2.1. Reagents

Sodium hyaluronate (HA) at high MW was kindly gifted by The Wave Innovation Group (Verona, Italy). Unless otherwise stated, all other chemicals and reagents were purchased from Merck (Milan, Italy) and were of analytical or cell-culture grade, as appropriate.

### 2.2. Cell Cultures and Treatments

The human synovial cell line SW982 was obtained from the American Type Culture Collection (ATCC, Rockville, MD, USA) and utilized for experiments between passages 4 and 10. Cells were maintained in 75 cm^2^ culture flasks using Dulbecco’s Modified Eagle Medium (DMEM), supplemented with 10% fetal bovine serum, 1% non-essential amino acids, 10 mM HEPES, 50 U/mL penicillin, and 50 µg/mL streptomycin. Cultures were incubated at 37 °C in a humidified atmosphere containing 5% CO_2_, with medium renewal every 48 h.

For the experiments, cells were seeded in 24-well plates at 6 × 10^4^ cells/cm^2^. After 24 h, they were treated with 10 ng/mL IL-1β (MedChemExpress, Monmouth Junction, NJ, USA) for an additional 24 h. Where indicated, cells were pre-treated for 1 h with HA, XAN, and/or EGCG at the specified concentrations. Stock solutions of XAN and EGCG were prepared in DMSO; upon dilution in DMEM, the final DMSO concentration never exceeded 0.1% (*v*/*v*).

### 2.3. Cell Viability

The cytotoxic effect of HA, XAN, and EGCG on SW982 synovial cells was evaluated using the MTT assay, a standard colorimetric method that assesses cell metabolic activity. This technique relies on the enzymatic reduction of the tetrazolium compound MTT (3-(4,5-dimethylthiazol-2-yl)-2,5-diphenyl tetrazolium bromide) into purple formazan crystals by mitochondrial dehydrogenases in viable cells. SW982 cells were seeded into 96-well plates (Corning Costar, Milan, Italy) at a concentration of 7.5 × 10^4^ cells/cm^2^ and allowed to adhere overnight. Subsequently, they were exposed to control conditions or treated with HA, XAN, or EGCG. After 24 h, the medium was gently aspirated and 4 µL of MTT solution (5 mg/mL) was added to each well. Following a 2 h incubation at 37 °C, the supernatant was removed, and the resulting formazan crystals were solubilized using 100 µL dimethyl sulfoxide (DMSO). The absorbance value at 575 nm, which characterizes the purple formazan, was measured with a microplate reader (LTek, INNO, Seongnam, Republic of Korea). The value of the control cells was considered to indicate 100% vitality [38]. Each experiment was repeated four times in triplicate.

### 2.4. Measurement of Intracellular Reactive Oxygen Species (ROS)

ROS generation was quantified by flow cytometry using dichloro-dihydro-fluorescein diacetate (DCFDA; Merck, Milan, Italy), as previously described [39]. Briefly, 0.5 µM DCFDA (Merck) was added to cells 30 min before the end of the treatment. After trypsinization, the cells were pelleted (2000× *g*, 4 °C, 5 min), washed in PBS, and resuspended in 400 µL PBS for analysis (CytoFLEX, Beckman Coulter, Brea, CA, USA). A minimum of 10,000 events/sample was acquired.

### 2.5. Nitric Oxide (NO) Determination

The level of NO released from the cells, present in the medium as nitrite, was assessed spectrophotometrically using the Griess assay (Thermo Fisher Scientific Inc., Waltham, MA, USA). Equal volumes (100 µL) of medium and Griess reagent (1% sulfanilamide in 5% phosphoric acid and 0.1% N-(1-naphthyl)ethylenediamine) were mixed and incubated for 10 min at room temperature. Absorbance was recorded at 540 nm (LTek, INNO) [40].

### 2.6. Combination Index (CI) Analysis

The Chou–Talalay method [41] was used to evaluate the combined effects of HA, XAN, and/or EGCG on ROS and NO levels. Fixed-ratio combinations of the individual compounds were prepared based on their respective IC_50_ values, maintaining a 1:1 potency ratio. These combinations were then serially diluted at ratios of 1:2, 1:2.66, 1:5, and 1:10 to assess their combined effects. The CI and the dose reduction index (DRI) were calculated using CompuSyn 1.0 software (ComboSyn, Paramus, NJ, USA). CI values <1, =1, or >1 indicate synergism, additivity, or antagonism, respectively [42].

### 2.7. ELISA

The release of the cytokines IL-6 (SEKB10395-5), IL-8 (SEK10098), IL-10 (SEKA10947-5), TNF-α (SEKA10602-5), and MMP-3 (SEK10467-5) into the culture medium was determined using an ELISA assay, according to the manufacturer’s instructions (Sino Biological, Inc., Beijing, China).

### 2.8. Western Blot Analysis

Following 24 h of treatment, approximately 6 × 10^6^ cells were harvested, rinsed twice with PBS, and resuspended in 250 µL of lysis buffer composed of 20 mM Tris-HCl (pH 7.6), 100 mM NaCl, 10 mM MgCl_2_, 2 mM PMSF, 0.5 mM DTT, and 2 mg/mL lysozyme, supplemented with a protease inhibitor cocktail (Roche Applied Science, Indianapolis, IN, USA, 11836170001). Cell disruption was carried out by sonication for 60 s in an ice-cooled water bath using a Labsonic LBS1-10 system (Falc Instruments srl, Treviglio, BG, Italy). The lysates were then subjected to ultracentrifugation at 100,000× *g* for 1 h at 4 °C. The resulting supernatant, representing the total protein fraction, was collected, and the protein content was quantified using the Bradford assay (Bio-Rad, Hercules, CA, USA). The prepared samples were then appropriately diluted to load 50 μg of protein per well onto the polyacrylamide gel (10% resolving gel with 4% stacking). Proteins were separated based on molecular weight by SDS-PAGE and transferred to a nitrocellulose membrane via electroblotting. The membrane was incubated for 1 h at room temperature with a diluted protein solution (5% skim milk) to block non-specific binding sites. The nitrocellulose membrane was then incubated overnight at 4 °C with primary antibodies against MMP-1 (AB-84794, Immunological Science (IS), Rome, Italy), MMP-3 (AB-84795, IS), phospho-Ser281-NF-κB p65 (AP-0418, IS), NF-κB p65 (AB-82033, IS), COX-2 (sc-19999, Santa Cruz Biotechnology (SCBT), Inc., Dallas, TX, USA), iNOS (sc-7271, SCBT), IκB- α (AB-10165, IS), p-IκB-α (BSM-60711r, Bioss Inc., Woburn, MA, USA), and Actin (sc-8432, SCBT), and then washed twice with TTBS (Tween20/Tris-buffered saline) to remove any unbound primary antibody. The membrane was subsequently incubated for 1 h at room temperature with a 1:2000 dilution of a secondary antibody (goat anti-mouse or goat anti-rabbit) conjugated with horseradish peroxidase (Merck KGaA, Darmstadt, Germany); it was then washed 5 times with TTBS to remove excess secondary antibody and developed using chemiluminescence (Amersham ECL RPN2106, Cytiva Europe GmbH, Freiburg, Germany). The chemiluminescent bands were evaluated with a C-Digit Blot Scanner (LI-COR, Lincoln, NE, USA) and band intensities were analyzed with LI-COR Image Studio 4.0 [43].

To evaluate the nuclear levels of the p65 subunit, nuclear extracts were prepared following the method described by Restivo et al. [40], with slight modifications. In brief, cells were lysed using a hypotonic buffer containing 10 mM HEPES-KOH (pH 7.9), 1.5 mM MgCl_2_, 10 mM KCl, 1 mM EDTA, 1% NP-40, 0.5 mM DTT, 1 mM PMSF, and 10 µg/mL aprotinin. Lysates were centrifuged at 2600× *g* for 3 min at 4 °C to separate the nuclear pellet. The pellets were then resuspended in nuclear lysis buffer consisting of 20 mM HEPES-KOH (pH 7.9), 10% glycerol, 420 mM NaCl, 1.5 mM MgCl_2_, 0.2 mM EDTA, 0.5 mM DTT, 1 mM PMSF, and 10 µg/mL aprotinin, followed by incubation on ice for 30 min. The nuclear lysates were clarified by centrifugation at 21,000× *g* for 10 min at 4 °C. Aliquots containing 50 µg of nuclear protein were then used to quantify p65 expression levels, as described previously. The data are reported as the mean ± standard deviation based on densitometric analysis of immunoreactive bands. The results, as reported in the captions, were normalized to actin, IκB- α, NF-κB, p65 or Lamin B1. A representative membrane was selected for each protein to assemble the figures (Figures S1 and S2).

### 2.9. Statistical Analysis

The results are expressed as the mean ± standard deviation (SD) of n separate experiments conducted in triplicate. Statistical comparisons were performed using one-way analysis of variance (ANOVA), followed by Tukey’s correction for multiple comparisons, using Prism 9.5.0 (GraphPad Software Inc., San Diego, CA, USA). In all cases, results with a *p*-value < 0.05 were considered statistically significant.

## 3. Results

### 3.1. Cytotoxicity of HA, XAN, and EGCG to SW982

**Figure 1:**
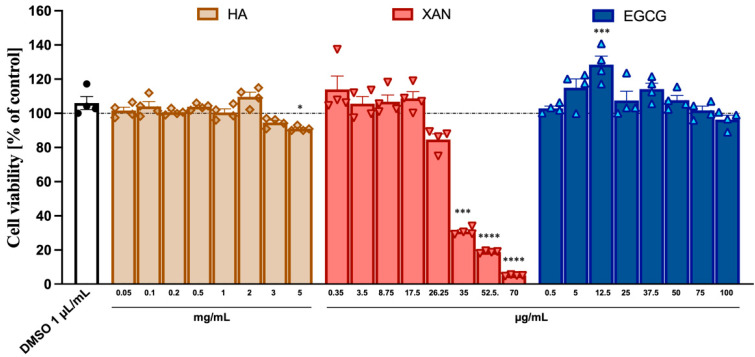
Cytotoxicity of HA, XAN, and EGCG to synoviocytes SW982 cells. Cell viability was assessed after 24 h treatment by MTT test. Values are mean ± SD of four separate experiments conducted in triplicate. * *p* < 0.05, *** *p* < 0.005, **** *p* < 0.001 vs. control DMSO. (ANOVA associated with Tukey’s test).

The potential cytotoxicity to SW982 synoviocytes of HA (from 0.05 to 5.0 mg/mL) and the phytochemicals XAN (from 0.35 to 70 μg/mL) and EGCG (from 0.5 to 100 μg/mL) was ascertained using the MTT assay. While HA and EGCG did not appear to exert any toxic effects even at the highest concentration used, XAN at concentrations > 20 μg/mL caused a dose-dependent reduction in cell viability (Figure 1).

### 3.2. Effect of HA in Combination with EGCG and/or XAN on IL-1 β-Induced ROS Production and NO Release in SW982 Cells

A common feature of inflamed tissues is the dysregulation of ROS and nitrogen species production. Intracellular ROS levels and NO release in the culture medium were measured in IL-1β-stimulated SW982 cells using flow cytometry with DCFDA as the fluorescent probe, and a spectrophometric Griess assay, respectively. In comparison with the control cells (vehicle alone), 24 h treatment of cells with IL1-1β (10 ng/mL) caused a significant overproduction of ROS and increase in nitrite in the medium.

**Figure 2:**
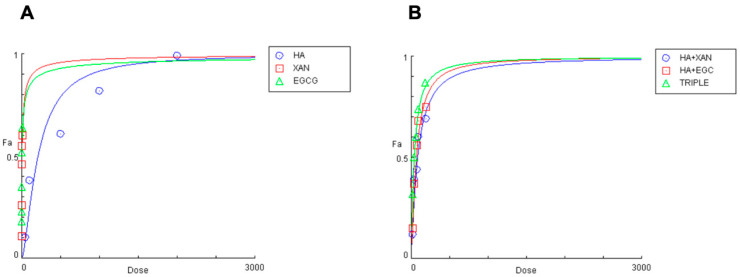
Effects of HA (0.05–2 mg/mL), EGCG (0.5–10 μg/mL), and XAN (0.30–15 μg/mL) (**A**), and of HA combined with XAN (20/0.6; 40/1.2; 75/2.25; 100/3; 200/6 μg/mL) or with EGCG (20/0.4; 40/0.8; 75/1.5; 100/2; 200/4 μg/mL), or the TRIPLE treatment (20/0.6/0.4; 40/1.2/0.8; 75/2.25/1.5; 100.0/3.0/2.0; 200/6.0/4.0 μg/mL) (**B**), on IL-1β-induced ROS production in SW982 cells.

Co-incubation with HA (0.05–2 mg/mL) and either XAN (0.30–15 μg/mL) or EGCG (0.5–10 μg/mL) resulted in a dose-dependent reduction in intracellular ROS levels (Figure 2A).

The concentrations of the biomolecules that achieved 50% inhibition of ROS production were 208 ± 9.851 μg/mL, 6.17 ± 0.51 μg/mL, and 4.59 ± 0.39 μg/mL (*n* = 3) for HA, XAN, and EGCG, respectively. The inhibitory effect of co-treatment with HA combined with XAN or EGCG, or with both the phytochemicals (TRIPLE), was then examined using various dilutions of an equipotent mixture of the components, in accordance with the Chou–Talalay method (Figure 2B) [41]. Table 1 reports the quantitative data illustrating the dose–response relationship for HA, XAN, and EGCG, individually or in combination, on IL-1β-induced ROS production in synoviocytes.

The concentration of HA required to inhibit half of the oxidative stress induced by inflammatory insult is reduced by 61% and 69% in the presence of XAN or EGCG, respectively, while a significantly greater reduction (80%, *p* < 0.005) is evident when glycosaminoglycan is added simultaneously with both phytochemicals. A clear synergistic effect (CI < 1) between HA and the phytochemicals is evident in all studied combinations at fa > 0.25. Interestingly, the CI values are smaller when HA is present with XAN plus EGCG, rather than with either one or the other (Table 1). Additionally, the dose reduction index (DRI) values indicate that the effective concentration of HA, in combination with XAN or EGCG, is reduced by approximately 2.5 to 3.5 times, while a more marked reduction, from 4.3 to 7 times, is evident when HA is added to the culture medium along with both XAN and EGCG (Table 1).

Similar results were obtained by analyzing the effect of the combination of HA with XAN and/or EGCG on NO release. Table 2 reports the quantitative data illustrating the dose–response relationships measured for the individual compounds and their combinations.

The concentrations of HA, XAN, and EGCG that cause 50% inhibition of NO production were calculated to be 222 ± 8.05 μg/mL, 5.01 ± 0.48 μg/mL, and 3.41 ± 0.29 μg/mL (*n* = 3), respectively. A clear synergistic effect with CI < 1 on nitrite production inhibition is evident for fa > 0.25. The greatest inhibitory effect is observed when HA is added simultaneously with the two phytochemicals, resulting in an 81% reduction in the IC50 of HA, the smallest CI values, and the highest DRI indexes.

Overall, our results, showing that HA exerts anti-inflammatory synergistic effects in combination with XAN or EGCG, demonstrate that the highest response is achieved when the three biomolecules are simultaneously present.

### 3.3. Effect of HA in Combination with XAN Plus EGCG on Cytokine and Metal Protease Release

**Figure 3:**
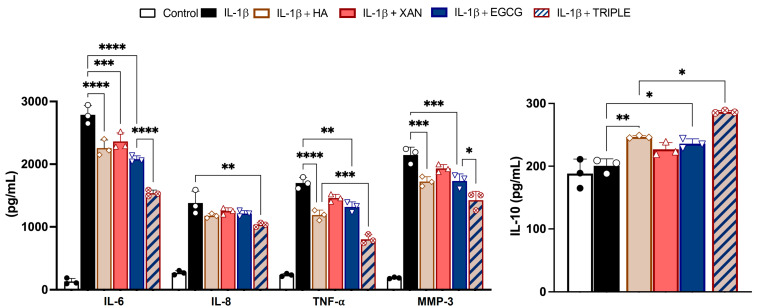
Effects of HA (200 μg/mL), XAN (6.2 μg/mL), EGCG (4.6 μg/mL), or HA in TRIPLE treatment (HA 200/XAN 6.2/EGCG 4.6 μg/mL) on release of IL-6, IL-8, IL-10, TNF-α, and MMP-3 in IL-1β-induced SW982 cells. Values are mean ± SD of three separate ELISA experiments conducted in triplicate. * *p* < 0.05, ** *p* < 0.01, *** *p* < 0.005, **** *p* < 0.001. (ANOVA associated with Tukey’s test).

The secretion of pro-inflammatory and anti-inflammatory cytokines and proteolytic enzymes from SW982 cells was measured by ELISA. Stimulation of synoviocytes with IL-1β for 24 h increased the levels of IL-6, IL-8, and TNF-α, as well as the metalloprotease enzyme MMP-3 (Figure 3), in the culture medium.

Co-treatment with TRIPLE, at concentrations corresponding to the respective IC50 values measured for ROS formation, significantly reduced the release of the hallmarks of the inflammatory process more effectively than co-treatment with the individual compounds. Specifically, TRIPLE decreased the IL-6 levels by 25% (*p* < 0.001) compared to EGCG, and decreased the TNF-α levels by 33% (*p* < 0.005) compared to HA, which were the most effective individual molecules in reducing these cytokines, respectively. Interestingly, incubation of the cells with IL-1β in the presence of HA, XAN, or EGCG resulted in the release of the anti-inflammatory cytokine IL-10. In this case, TRIPLE was also more effective, increasing the IL-10 level by approximately 17% compared to the most active compound, i.e., HA. With respect to IL-8, while individual compounds did not significantly inhibit cytokine release when compared to cells stimulated with IL-1β alone (*p* > 0.05), a significant decrement (about 25%, *p* < 0.01) was observed with TRIPLE (Figure 3).

Overall, these results indicate that the combined action of HA with both the phytochemicals, XAN plus EGCG, produces a higher response rate to pro-inflammatory injury when compared to the single compounds.

### 3.4. Effect of HA in Combination with XAN Plus EGCG on Expression of Pro-Inflammatory Enzymes

**Figure 4:**
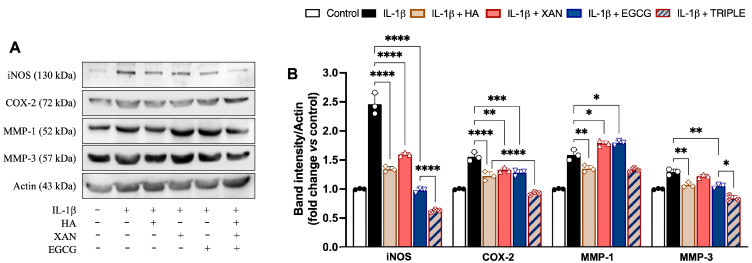
Effects of HA (200 μg/mL), XAN (6.17 μg/mL), EGCG (4.60 μg/mL), or HA with XAN plus EGCG (TRIPLE) on protein levels of iNOS, COX-2, MMP-1, and MMP-3 in IL-1β-induced SW982 cells. (**A**) Representative images of analyzed proteins. (**B**) Densitometric analysis of iNOS, COX-2, MMP-1, and MMP-3 normalized for actin. Values are means ± SD of bands’ densitometry of three independent experiments with comparable results. * *p* < 0.05, ** *p* < 0.01, *** *p* < 0.005, **** *p* < 0.001. (ANOVA associated with Tukey’s test).

The levels of inflammatory enzymes such as iNOS, COX-2, MMP-1, and MMP-3 were measured in SW982 cells by Western blot analysis. As expected, stimulation with IL-1β induced a net increase in the levels of all the evaluated proteins compared to the control (Figure 4).

Except for MMP-1, co-incubation of the cells with TRIPLE more effectively reduced the IL-1β-dependent increase in protein amounts in comparison to the individual compounds. Specifically, TRIPLE significantly inhibited the iNOS and MMP-3 protein levels by 34% (0.001) and 20% (*p* < 0.05), respectively, in comparison to EGCG, and decreased the COX-2 levels by 21% (*p* < 0.001) in comparison to HA. For the MMP-1 levels, no significant change was observed between TRIPLE and HA, which was the compound causing the greatest inhibition (16%, *p* < 0.01), compared to cells stimulated with IL-1β alone. EGCG and XAN, when individually present, appeared to slightly but significantly (*p* < 0.05) increase the 1L-1β induced protein level.

### 3.5. Effect of HA in Combination with XAN Plus EGCG on Activation of Redox-Dependent Transcription Factor NF-κB

**Figure 5:**
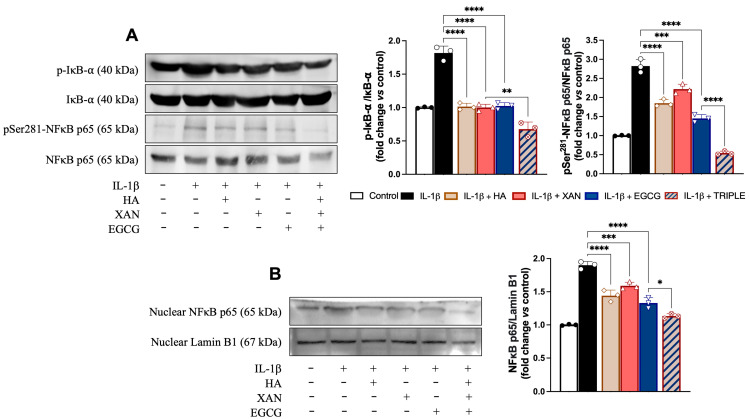
Effects of HA (200 μg/mL), XAN (6.17 μg/mL), EGCG (4.60 μg/mL), or TRIPLE on protein levels of p-IkB, p-p65, and nuclear p65 in IL-1β-induced SW982 cells. (**A**) Representative images of analyzed proteins and densitometric analysis of p-IkB/IkB and p-65/p65 ratios. (**B**) Representative images of analyzed proteins and densitometric analysis of nuclear traslocation of p65. Values are means ± SD of bands’ densitometry of three independent experiments with comparable results. * *p* < 0.05, ** *p* < 0.01, *** *p* < 0.005, **** *p* < 0.001. (ANOVA associated with Tukey’s test).

The activation of NF-κB (p65 and p50) depends on the phosphorylation and subsequent degradation of IκB, an endogenous inhibitor that binds to NF-κB, keeping it in the cytoplasm. Upon release from IκB, NF-κB, following phosphorylation, translocates to the nucleus, where it binds to specific NF-kB DNA response elements and initiates expression of inflammatory enzymes and other mediators. Using Western blot analysis, we investigated NF-κB activation pathway proteins to attempt to explore the mechanism underlying the cooperation between HA and EGCG plus XAN in alleviating the inflammatory response in synoviocytes. In our conditions, treatment of SW982 cells with IL-1β increased both the p-IkB/IkB ratio and the p-p65/p65 ratio, while the presence of p-p65 in nuclei was evident (Figure 5). Co-treatment of the cells with HA, XAN, or EGCG alone significantly reduced the levels of phosphorylated IkB and p65 in favor of the non-phosphorylated forms (Figure 5A). In addition, nucleus translocation of p-p65 was also significantly (*p* < 0.0001) inhibited (Figure 5B). Interestingly, in comparison with the individual biomolecules, TRIPLE more strongly reduced the ratio between the phosphorylated and non-phosphorylated forms of the proteins (*p* < 0.005), bringing the values below those of the control, and completely prevented the translocation of p-65 into the nucleus (Figure 5).

## 4. Discussion

Intra-articular HA injection, termed viscosupplementation, is recognized as a safe and first-choice treatment of arthropathy as it both enhances intra-articular viscoelasticity and alleviates the inflammatory status of the joints [21,22]. The development of combination targeted therapies designed to improve therapeutic efficacy while minimizing side effects is a key focus in both basic research and clinical trials. Mimicking in vitro a rheumatoid arthritis microenvironment with synoviocytes exposed to IL-1β, the present study shows that HA can synergistically interact with XAN and/or EGCG to target NF-κB and attenuate the inflammatory cell response. The most pronounced effect is observed when all three biomolecules are administered simultaneously. The administration of XAN and EGCG alone led to significant attenuation of the inflammatory response, confirming their individual efficacy [29,30,31,32,33,34,35,36]. However, their combined use with HA led to an even more marked reduction in cell oxidative/nitrosative stress and in NF-κB activation, which supports a true synergistic interaction. Our findings indicate that the interaction between HA and the natural compounds does not merely counteract the effects of XAN and EGCG, but rather potentiates their action, highlighting the potential of these combinations in targeting inflammatory pathways.

Owing to its high biocompatibility and affinity for the CD44 receptor, overexpressed in synoviocytes, HA can serve as an effective carrier for targeted drug delivery in inflamed joints. Lee et al. developed HA–EGCG conjugates that exhibited enhanced anti-proliferative and anti-inflammatory activity in vitro compared to free EGCG. These conjugates significantly suppressed synoviocyte proliferation and IL-6 secretion, and preferentially accumulated in inflamed joints in a collagen-induced arthritis rat model, supporting their targeted delivery and therapeutic potential [44]. In a separate study, HA–curcumin nanomicelles synthesized via ester linkage demonstrated dual functionality by inhibiting pro-inflammatory cytokine production in synovial cells and promoting chondrocyte proliferation [45]. Jin et al. formulated an injectable HA–gelatin–EGCG hydrogel designed to attenuate inflammation and support cartilage repair. In vitro, the hydrogel protected chondrocytes from IL-1β-induced damage, while in vivo studies in an osteoarthritis model confirmed its ability to reduce cartilage degradation and promote chondrogenic regeneration [46]. Collectively, these findings underscore the utility of HA–phytochemical systems as multifunctional therapeutic platforms in RA, offering advantages in targeted delivery, anti-inflammatory activity, and joint-tissue preservation.

NF-κB is a key nuclear transcription factor in orchestrating the inflammatory response triggered by IL-1β, and its activation is regulated by an intricate network of signaling pathways [37]. The rationale behind the synergistic effect of HA in combination with EGCG and XAN described in this study may lie in their ability to inhibit NF-kB stimulation through distinct mechanisms. This results in a more rapid and effective response compared to the use of the individual compounds alone.

Upon binding of the pro-inflammatory cytokine IL-1β, the membrane-bound IL-1 receptor recruits various adaptor proteins, leading to the activation of a MyD88-dependent signaling complex that culminates in the activation tumor necrosis factor receptor molecule-associated factor-6 (TRAF-6). TRFA-6 stimulates the NF-kB activation pathway, allowing the transcription factor to migrate to the nucleus, where it regulates the expression of genes involved in the inflammatory response, such as *iNOS*, *MMPs*, and *COX-2* [47]. ROS also play a significant role in NF-κB activation. ROS can directly activate IkB kinase, responsible for the phosphorylation and subsequent degradation of the IκB complex, or promote the stimulation of upstream signaling molecules, such as protein kinase C and MAPKs, which, in turn, activate NF-κB [48]. Moreover, recent studies have revealed the crosstalk and interaction between NF-κB and other signaling pathways, such as PI3K/Akt and Wnt/β-catenin [37]. Within this intricate and complex regulatory network, HA, XAN, and EGCG may influence the molecular signaling involved in NF-κB transcription factor activation at various different levels.

In rheumatoid synovial fibroblasts, high-molecular-weight HA plays a well-established anti-inflammatory role, primarily through its interaction with adhesion molecules. Binding of HA to the cell-surface receptor cluster of differentiation 44 appears to be involved in the suppression of MMP-1 production in TNF-α or IL-1β-stimulated synoviocites [49]. In these cells, it has also been reported that HA suppresses IL-1β-induced MMP synthesis by downregulating NF-kB phosphorylation and p38 MAPK activation through its interaction with ICAM-1 [50]. Moreover, Lee et al. demonstrated that treatment of inflamed primary synovial cells with HA resulted in the inhibition of NF-κB nuclear translocation, associated with downregulation of the endoplasmic reticulum stress chaperone GRP78, highlighting anti-inflammatory activity of the biomolecule under IL-1β-induced stimulation of abnormal oxidative stress [51].

In synovial fibroblasts, EGCG has been reported to reduce IL-1β-induced production of chemokines and MMP-2. Evaluation of signaling events revealed that EGCG preferentially blocked the phosphorylation of PKCδ and inhibited the activation and nuclear translocation of NF-kB [31]. Other studies on human chondrocytes provide direct evidence of the role of EGCG in inhibiting IL-1β-induced degradation of endogenous IκBα through downregulation of TRAF-6 expression, a crucial protein in the IL-1β-activated signal transduction pathway. In addition, the JNK-MAPK pathway is also inhibited by EGCG [30].

The protective effects of XAN have been extensively investigated in various inflammation models, both in cultured cells and in animals [52]. The anti-inflammatory action of XAN is postulated to occur through several mechanisms. It has been reported that the flavonoid can interfere in the recruitment of coreceptor proteins that are essential for the formation of the MyD88 signaling complex [53]. In inflamed chondrocytes, it has been clearly demonstrated that XAN can attenuate the pro-inflammatory activity of NF-κB through Nrf2 activation [33]. Our study demonstrates, for the first time, that in synoviocytes, XAN can inhibit the IL-1β-induced signaling cascade by blocking the NF-κB activation pathway. In this context, it has been reported that XAN can bind covalently to Cys residues of IKK, the kinase for the inhibitory subunit of NF-κB, as well as to Cys residues of the p65 subunit of NF-κB [54].

Since EGCG and XAN are phytochemicals with potent reducing properties, their capacity to inhibit the activation of the redox-sensitive transcription factor NF-κB by restoring the redox equilibrium disrupted by the cytokine IL-1β [55] should also be considered. In our study, we observed that both EGCG and XAN, when added individually to IL-1 β-stimulated SW982 cells, led to a modest upregulation of MMP-1 expression. This finding contrasts with the existing literature, where both phytochemicals have been reported to inhibit MMP-1 expression in various cell types, such as fibroblasts and keratinocytes, under different inflammatory stimuli. Specifically, EGCG has been shown to suppress MMP-1 expression induced by TNF-α in human dermal fibroblasts by inhibiting the MAPK/ERK signaling pathway and reducing AP-1 transcriptional activity [56]. Similarly, XAN has demonstrated the ability to inhibit MMP-1 activity and promote the expression of extracellular matrix components in skin fibroblasts [57]. The discrepancy between our findings and those reported in the literature may be attributed to cell-type-specific responses to these compounds. It is plausible that the regulatory effects of EGCG and XAN on MMP-1 expression are context-dependent, varying according to the cellular microenvironment and the specific signaling pathways activated in different cell types. While our findings indicate that the effects of HA, EGCG, and XAN in IL-1β-stimulated synoviocytes converge on the inhibition of the NF-κB signaling pathway, other molecular pathways may also be modulated by these biomolecules and contribute to the observed anti-inflammatory effects when used in combination. This hypothesis warrants further investigation.

Our study offers several strengths, including the use of a physiologically relevant in vitro model of rheumatoid arthritis, in which synoviocytes were stimulated with IL-1β to mimic the inflammatory joint microenvironment. The evaluation of defined combinations of Ha, EGCG, and XAN allowed us to demonstrate, for the first time, a synergistic anti-inflammatory effect mediated by the concurrent inhibition of NF-κB activation and reduction of oxidative/nitrosative stress. These findings contribute to the growing body of evidence supporting the therapeutic utility of HA–phytochemical systems in inflammatory joint disorders. However, our study also presents limitations. The data are limited to a single cell line and an acute stimulation model, which may not fully replicate the complexity of the in vivo RA microenvironment. Moreover, although our results suggest a synergistic mechanism, additional mechanistic studies are required to dissect the specific molecular interactions and signaling cascades involved. The impact of these compounds on other relevant cell types, such as macrophages and chondrocytes, also remains to be explored. Future research should aim to validate these findings in animal models of arthritis, evaluate the pharmacokinetics and biocompatibility of injectable HA-based formulations, and assess long-term efficacy and safety.

## Figures and Tables

**Table 1:** Dose–response association of HA, XAN, and EGCG, alone or in combination, with IL-1β-induced ROS production in SW982 cell line.

Compound	IC_50_	CI Values			DRI Value			DRI Value			DRI Value		
					Combo with XAN			Combo with EGCG			Combo with XAN Plus EGCG		
	mg/mL	*f*_α0.25_	*f*_α0.5_	*f*_α0.75_	*f*_α0.25_	*f*_α0.5_	*f*_α0.75_	*f*_α0.25_	*f*_α0.5_	*f*_α0.75_	*f*_α0.25_	*f*_α0.5_	*f*_α0.75_
HA	208.66				3.27	2.62	2.11	3.77	3.24	2.78	4.34	5.56	7.12
XAN	6.17												
EGCG	4.59												
HA:XAN	81.89	1.00	0.77	0.69									
(33:1)													
HA:EGCG	65.80	1.21	0.62	0.46									
(50:1)													
HA:XAN:EGCG	39.40	0.88	0.52	0.39									
TRIPLE (50:1.5:1)													

DRI, dose reduction index; a greater DRI value indicates a greater dose reduction for a given therapeutic effect.

**Table 2:** Dose–response association of HA, XAN, EGCG, alone or in combination, with IL-1β-induced NO release in SW982 cell line.

Compound	IC_50_	CI Values			DRI Value			DRI Value			DRI Value		
					Combo with XAN			Combo with EGCG			Combo with XAN Plus EGCG		
	mg/mL	*f*_α0.25_	*f*_α0.5_	*f*_α0.75_	*f*_α0.25_	*f*_α0.5_	*f*_α 0.75_	*f*_α0.25_	*f*_α0.5_	*f*_α0.75_	*f*_α0.25_	*f*_α0.5_	*f*_α0.75_
HA	222.64				2.11	3.08	4.51	2.31	2.87	3.54	3.81	5.77	8.75
XAN	5.08												
EGCG	3.40												
HA:XAN	74.28	1.04	0.75	0.54									
(33:1)													
HA:EGCG	79.44	1.31	0.85	0.57									
(50:1)													
HA:XAN:EGCG	40.51	1.01	0.62	0.39									
TRIPLE (50:1.5:1)													

DRI, dose reduction index; a greater DRI value indicates a greater dose reduction for a given therapeutic effect.

## Data Availability

Data is contained within the article and Supplementary Materials.

## Supplementary Materials

Supporting information can be downloaded at https://www.mdpi.com/article/10.3390/antiox14060713/s1. Figure S1: Complete blot images in Figure 4. Figure S2: Complete blot images in Figure 5.

## Abbreviations

The following abbreviations are used in this manuscript:

HA	hyaluronic acid
XAN	Xanthohumol
EGCG	Epigallocatechin-3-O-Gallate
IL-1β	interleukin-1beta
ROS	reactive oxygen species
NO	nitric oxide
TRIPLE	combination of HA, XAN, and EGCG
COX-2	cyclooxygenase-2
iNOS	nitric oxide synthetase
MMPs	matrix metalloproteases
DMEM	Dulbecco’s modified Eagle’s medium
DCFDA	dichloro-dihydro-fluorescein diacetate
CI	combination index
*fa*	effect fraction
DRI	dose reduction index
TTBS	Tween20/Tris-buffered saline
SD	standard deviation
ANOVA	one-way analysis of variance
