# Supplementary material for: Cooperative Interaction of Hyaluronic Acid with Epigallocatechin-3-O-gallate and Xanthohumol in Targeting the NF-κB Signaling Pathway in a Cellular Model of Rheumatoid Arthritis
Source: Antioxidants (Basel). 2025 Jun 11;14(6):713. doi: 10.3390/antiox14060713 (PMC12189393; doi:10.3390/antiox14060713)

Cooperative Interaction of Hyaluronic Acid with Epigallocatechin-3-O-gallate and Xanthohumol in Targeting the NF-κB Signaling Pathway in a Cellular Model of Rheumatoid Arthritis

Francesco Longo, Alessandro Massaro, Manuela Mauro, Mario Allegra, Vincenzo Arizza, Luisa Tesoriere and Ignazio Restivo *

Supplementary Materials

General consideration

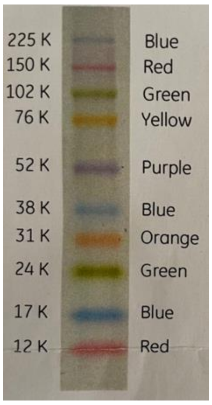

To avoid wasting large volumes of antibodies and developing solution, the membranes were trimmed at the upper and lower edges based on the full-range marker, which provides a colorimetric reference for the proteins of interest. Small cuts on the left/right side of the membrane correspond to the molec ular weights indicated by the marker. If these cuts are not visible, it is because the antibody has bound with extreme specificity, resulting in a membrane that is too clean to distinguish them.

1: Control, 2: IL-1β, 3: IL-1β + HA, 4: IL-1β + XAN, 5: IL-1β + EGCG, 6: IL-1β + TRIPLE.

Supplementary Figure S1. Complete blot images in Figure 4.

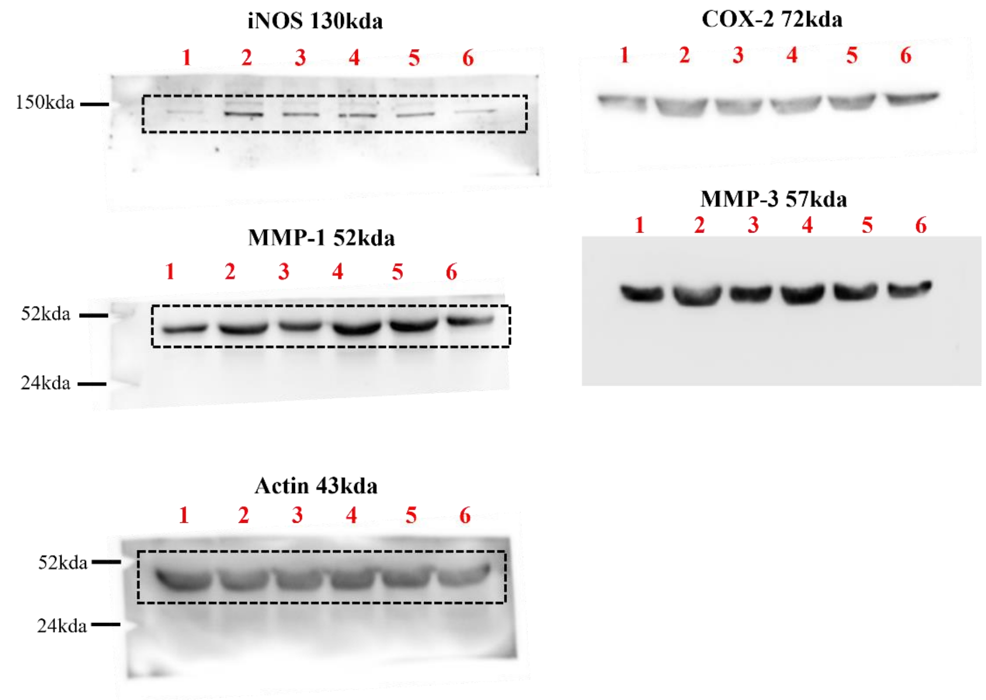

Supplementary Figure S2. Complete blot images in Figure 5.

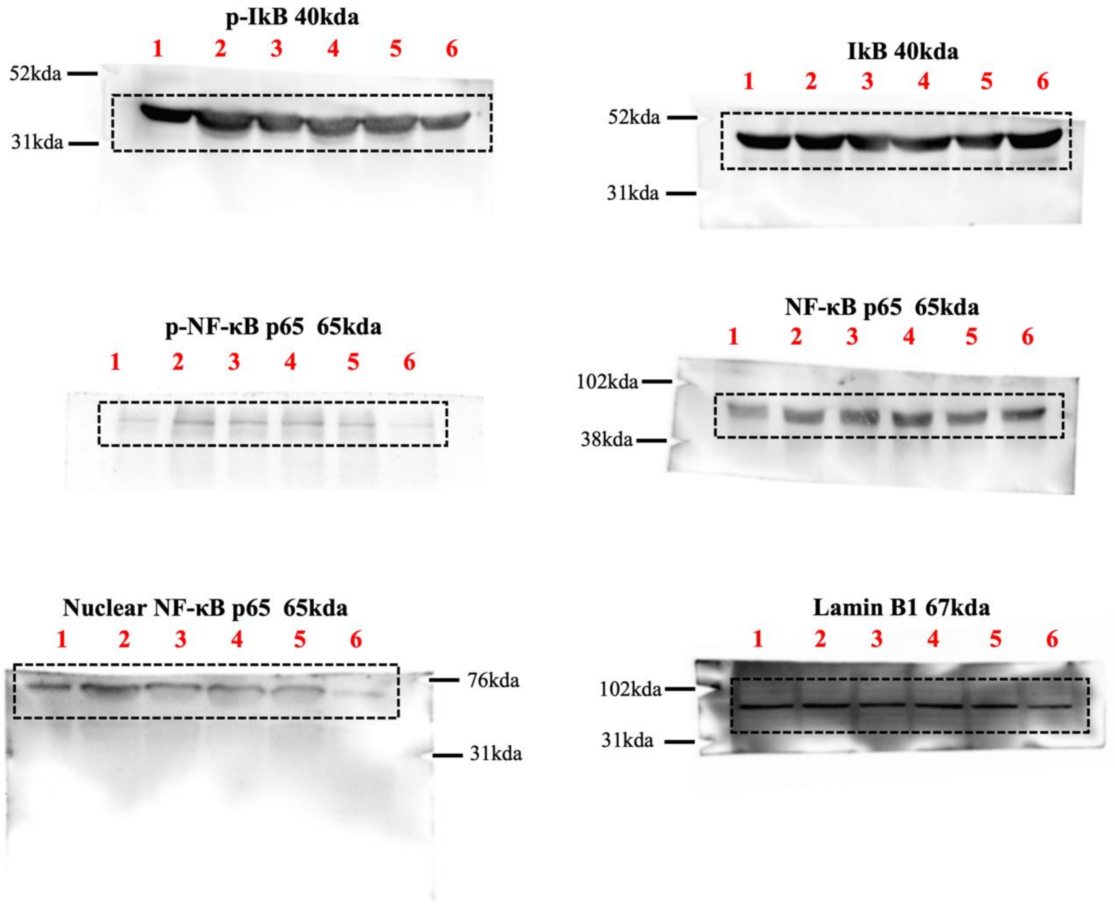

Supplement: Supplementary file 1 [file antioxidants-14-00713-s001.zip › antioxidants-3624732-supplementary.pdf]
